# Natural Products in Epilepsy Treatment: From Traditional Medicine Towards Computational Drug Discovery

**DOI:** 10.3390/cimb48050483

**Published:** 2026-05-06

**Authors:** Muhammad Yasir, Jin-Hee Han, Jongseon Choe, Wanjoo Chun

**Affiliations:** 1 Department of Pharmacology, Kangwon National University School of Medicine, Chuncheon 24341, Republic of Korea; yasir.khokhar1999@gmail.com; 2 Department of Medical Environmental Biology and Tropical Medicine, Kangwon National University School of Medicine, Chuncheon 24341, Republic of Korea; han.han@kangwon.ac.kr; 3 Department of Microbiology and Immunology, Kangwon National University School of Medicine, Chuncheon 24341, Republic of Korea; jchoe@kangwon.ac.kr

**Keywords:** epilepsy, natural products, antiepileptic drug discovery, GABA modulation, computational drug design, virtual screening, blood–brain barrier penetration

## Abstract

Epilepsy affects approximately 50 million people worldwide, with nearly one-third of patients experiencing drug-resistant seizures despite available antiepileptic drugs (AEDs). Natural products remain an important source of bioactive scaffolds for drug discovery, offering diverse chemical structures capable of modulating key pathological pathways in epilepsy. This review examines major classes of natural compounds, including alkaloids, flavonoids, terpenoids, and phenolic compounds, and their activity against validated targets such as GABAergic and glutamatergic systems, voltage-gated ion channels, and neuroinflammatory pathways. Advances in computational drug discovery have significantly accelerated the identification and optimization of these compounds. Approaches such as virtual screening, molecular docking, molecular dynamics simulations, and machine learning models, particularly graph neural networks (GNNs), enable the efficient prediction of compound target interactions, binding stability, and pharmacokinetic properties, including blood–brain barrier (BBB) penetration and ADMET profiles. These methods support the prioritization and rational modification of natural product leads from large chemical libraries. Notable clinical approval of cannabidiol (Epidiolex) highlights the translational potential of natural product-based therapeutics. However, challenges such as limited bioavailability, pharmacokinetic constraints, and variability in natural sources continue to hinder development. This review provides an integrated perspective on natural product scaffolds, their molecular targets, and the computational strategies driving their advancement toward novel antiepileptic therapies.

## 1. Introduction

### 1.1. Epilepsy: Clinical Overview and Treatment Challenges

Epilepsy is one of the most common chronic neurological disorders, characterized by recurrent and unprovoked seizures caused by abnormal electrical activity in the brain [1,2]. It affects approximately 50 million people worldwide, with nearly 2.4 million new cases reported annually. The disorder occurs across all age groups and populations, although its incidence is higher in low- and middle-income countries where access to adequate healthcare and treatment remains limited [3,4]. Antiepileptic drugs (AEDs) remain the primary strategy for epilepsy management. Over the past decades, AED development has progressed through distinct generations with increasingly refined mechanisms and safety profiles. First-generation agents, such as phenytoin, carbamazepine, and valproic acid, primarily modulate voltage-gated sodium/calcium channels and enhance GABAergic inhibition, but are frequently associated with significant adverse effects, including cognitive impairment, sedation, dizziness, and hepatotoxicity [5,6,7]. Second- and third-generation AEDs, including lamotrigine, levetiracetam, topiramate, and more recently brivaracetam, perampanel, and cenobamate, act through a broader repertoire of mechanisms such as presynaptic SV2A modulation, AMPA-receptor blockade, and calcium-channel inhibition, often with improved tolerability and fewer drug–drug interactions. This evolution reflects a deliberate shift from broad-spectrum ion-channel modulation toward more targeted, pathophysiology-based interventions, underpinned by advances in preclinical models and clinical trial design [8,9,10,11,12,13]. Despite these advances, approximately one-third of people with epilepsy develop drug resistant epilepsy (DRE), defined by the International League Against Epilepsy (ILAE) as the failure to achieve sustained seizure freedom after adequate trials of at least two appropriately selected and tolerated AED regimens, used alone or in combination. This definition emphasizes not only the number of drugs but also correct seizure-type matching, adequate dosing, and adherence, thereby helping to distinguish true pharmacoresistance from pseudo-resistance. DRE is associated with markedly reduced quality of life, higher rates of injury and sudden unexpected death in epilepsy (SUDEP), and increased socioeconomic burden [11,14].

Adverse effects of AEDs differ meaningfully between first-generation and newer agents. Older AEDs are more commonly linked to cognitive slowing, significant sedation, long-term bone-health risks, and teratogenicity, particularly with valproate during pregnancy. In contrast, many newer AEDs exhibit more favorable cognitive and neuropsychiatric profiles, although they are not free of side effects; for example, weight-neutral or weight-loss-prone agents (e.g., topiramate) can cause metabolic and cognitive complaints, while others (e.g., levetiracetam) may induce behavioral changes or mood disturbances. The narrower therapeutic windows and potential for drug–drug interactions in older AEDs further complicate polypharmacy, especially in older adults and patients with comorbidities [15,16]. 

### 1.2. Natural Products in Drug Discovery: From Traditional Knowledge to Modern Lead Discovery

Natural products have long served as important sources for pharmaceutical development, providing chemically diverse and biologically active compounds shaped by millions of years of evolutionary selection. Traditional medicine systems across different cultures have historically employed plant-derived remedies for seizure disorders, offering valuable ethnopharmacological knowledge that continues to guide modern drug discovery [17,18,19,20]. Analyses of drug development trends indicate that nearly half of the drugs approved between 1981 and 2014 were natural products, natural product derivatives, or compounds inspired by natural product scaffolds, highlighting their enduring contribution to therapeutic innovation [21,22,23,24].

In the field of epilepsy, the influence of natural products is well-recognized. Early AEDs such as phenytoin were structurally inspired by natural product frameworks [25,26], while more recently, cannabidiol (CBD), a phytocannabinoid derived from *Cannabis sativa*, was approved as Epidiolex for the treatment of Dravet syndrome and Lennox–Gastaut syndrome, representing a significant milestone in natural product-based antiepileptic therapy [27,28]. Natural products offer several advantages as drug discovery leads. They possess remarkable structural diversity, including complex three-dimensional architectures, multiple stereocenters, and diverse functional groups that are rarely achieved through conventional synthetic chemistry [29,30,31]. 

In addition, their evolutionary optimization often results in strong biological activity, target selectivity, and favorable pharmacokinetic properties. Importantly, many natural products act on multiple molecular targets, which may be beneficial for complex disorders such as epilepsy that involve interconnected mechanisms, including neurotransmitter imbalance, oxidative stress, neuroinflammation, and neuronal damage. This polypharmacological profile can enhance therapeutic efficacy by modulating several pathways simultaneously, potentially overcoming the limitations of single-target agents in heterogeneous disease states. However, this broad target engagement also carries the risk of off-target effects and idiosyncratic toxicity, particularly when key pharmacokinetic, metabolic, or safety-profile determinants are not fully characterized. For example, certain plant-derived compounds can interact with cytochrome P450 enzymes or transporters, leading to unpredictable drug–drug interactions or organ-specific toxicity, underscoring the need for rigorous preclinical and clinical evaluation before therapeutic deployment [32,33]. Thus, while multi-target natural products represent promising leads for antiepileptic drug discovery, their development must be accompanied by the careful optimization of selectivity and safety to harness their therapeutic potential while minimizing unintended adverse consequences.

### 1.3. The Computational Revolution in Natural Product Drug Discovery

The integration of computational methodologies has significantly advanced natural product-based drug discovery in recent decades [34,35]. Traditional strategies, such as bioassay-guided fractionation of crude extracts, although historically successful, are labor-intensive and limited in throughput. In contrast, modern computational approaches enable the rapid screening of extensive natural product libraries, prediction of pharmacological properties, and rational prioritization of promising candidates prior to experimental validation [34,35,36,37,38]. Structure-based virtual screening, particularly molecular docking, facilitates the evaluation of large compound libraries against therapeutic targets to identify potential binders [39,40]. In parallel, machine learning techniques, including graph neural networks (GNNs), allow the prediction of key molecular properties such as target affinity, blood–brain barrier (BBB) permeability, metabolic stability, and toxicity directly from molecular structures [41,42]. Molecular dynamics simulations further provide atomistic insights into protein–ligand interactions, supporting the refinement and optimization of candidate molecules [43,44,45]. These computational strategies are especially valuable for central nervous system drug discovery, where properties such as BBB permeability and favorable ADMET profiles are essential. Early prediction of these parameters helps prioritize compounds with higher therapeutic potential while reducing time and resource expenditure. The combination of computational screening with ethnopharmacological knowledge therefore represents an efficient strategy for identifying bioactive natural products with potential therapeutic applications.

## 2. Mechanisms of Epileptogenesis and Potential Targets

### 2.1. Pathophysiology of Epilepsy

Epileptogenesis is a multifactorial mechanism in which normal brain tissue acquires the ability to generate spontaneous recurrent seizures through interconnected molecular, cellular, and network level alterations [46,47,48]. Central to this process is excessive neuronal excitability and hyper synchronization, driven primarily by an imbalance between excitatory and inhibitory signaling [49,50]. Ion channels serve a critical role in regulating neuronal activity. Dysfunction of voltage-gated sodium channels promotes repetitive high-frequency firing, a hallmark of seizure activity, and forms the basis for the mechanism of several AEDs such as phenytoin, carbamazepine, and lamotrigine [51,52]. Voltage-gated sodium channels, particularly T-type channels, contribute to burst firing in absence seizures, while other subtypes regulate neurotransmitter release and excitotoxicity. In contrast, impaired function of voltage-gated potassium channels reduces repolarization capacity, further enhancing neuronal hyperexcitability [9,53,54,55,56,57,58].

**Figure 1:**
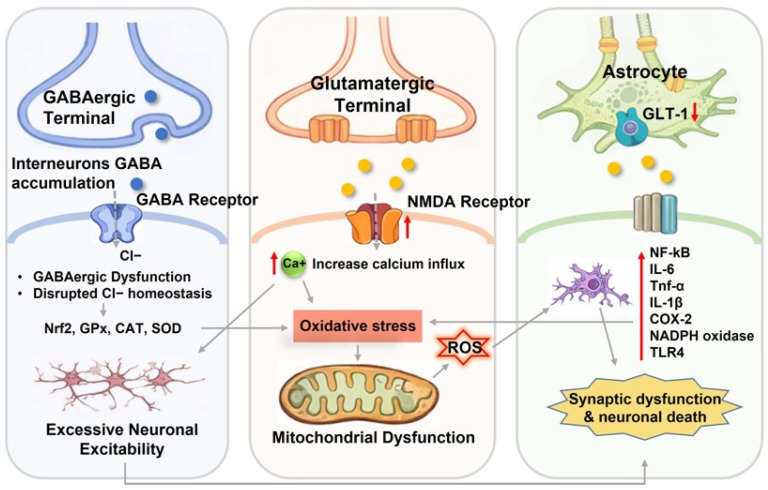
Mechanistic overview of epileptogenesis highlighting neurotransmitter imbalance, oxidative stress, and neuroinflammation.

In addition to ion channel dysfunction, disruption of the balance between γ-aminobutyric acid (GABA) and excitatory glutamate signaling is a feature of epileptogenesis [59,60,61,62,63]. GABAergic impairment may arise from reduced interneuron activity, altered receptor function, or increased degradation via GABA transaminase (GABA-AT). Inhibition of this enzyme increases synaptic GABA levels, highlighting its therapeutic relevance. Conversely, excessive glutamatergic activity, particularly through NMDA and AMPA receptors, promotes calcium influx, excitotoxicity, and seizure propagation (Figure 1) [64].

Seizure activity also induces oxidative stress through mechanisms such as excessive calcium influx and mitochondrial dysfunction, leading to the accumulation of reactive oxygen species (ROS) [65,66,67]. These reactive species cause lipid peroxidation, protein oxidation, and DNA damage, contributing to neuronal injury and progression of epileptogenesis [68,69]. Mitochondrial impairment further exacerbates this process by disrupting ATP production and calcium buffering, creating a cycle of metabolic dysfunction [70,71,72].

Neuroinflammation represents another critical component, acting both as a consequence and a driver of seizure activity. Activation of microglia and astrocytes results in the release of pro-inflammatory mediators, including Interleukin-1β (IL-1β), Interleukin-6 (IL-6), and Tumor Necrosis Factor-α (TNF-α), which enhances excitatory transmission, impairs inhibitory signaling, and disrupts BBB integrity [73,74,75,76,77,78,79]. These effects are mediated through key pathways such as NF-κB, COX-2, and NLRP3 inflammasome [80,81], further contributing to neuronal dysfunction. Furthermore, recurrent seizures lead to progressive neurodegeneration and structural reorganization, particularly within the hippocampus. Neuronal loss, mossy fiber sprouting, and aberrant neurogenesis promote the formation of recurrent excitatory circuits that sustain seizure activity [82,83]. Hippocampal sclerosis, a common pathological feature of temporal lobe epilepsy, exemplifies these changes [84,85].

### 2.2. Molecular Targets for Natural Products

Based on the underlying mechanisms of epileptogenesis, several molecular targets provide rational intervention points for natural product-based antiepileptic therapies. Voltage-gated ion channels remain central targets, including sodium (Nav1.1, Nav1.2, Nav1.6), calcium (T-type, L-type, N-type, and P/Q-type), and potassium channels (Kv7/KCNQ M-channels, BK, and K_ATP), where modulation can reduce neuronal hyperexcitability and abnormal synaptic transmission [86,87,88]. Enhancement of inhibitory neurotransmission through GABAergic pathways is another validated strategy, encompassing positive allosteric modulation of GABA_A_ and GABA_B_ receptors, inhibition of GABA-AT, activation of glutamic acid decarboxylase, and blockade of GABA transporters to increase synaptic GABA availability.

Excessive glutamatergic signaling also represents a key therapeutic axis. Selective modulation of NMDA receptor subunits, non-competitive antagonism of AMPA receptors, targeting of kainate receptors, and activation of group II/III metabotropic glutamate receptors may attenuate excitotoxicity while minimizing adverse effects [89,90]. In addition to neurotransmission, neuroinflammatory pathways, including NF-κB signaling, cyclooxygenase-2, pro-inflammatory cytokine receptors (e.g., IL-1β and TNF-α), and the NLRP3 inflammasome, contribute to seizure propagation and epileptogenesis and thus constitute important intervention points. Oxidative stress-related mechanisms, such as activation of the Nrf2 antioxidant pathway, direct ROS scavenging, and preservation of mitochondrial function, further expand the therapeutic landscape [91,92,93].

Given the multifactorial nature of epilepsy, natural products with multi-target activity may offer distinct advantages by simultaneously modulating ion channels, neurotransmitter systems, inflammatory mediators, and oxidative stress pathways. Such pleiotropic mechanisms may not only improve symptomatic seizure control, but also contribute to neuroprotection and potential disease modification.

## 3. Mechanistic Insights of Natural Products in Epilepsy

Natural products represent a chemically diverse reservoir of bioactive molecules capable of modulating multiple targets involved in epileptogenesis. Unlike conventional AEDs, which often act through single mechanisms, natural compounds typically exhibit polypharmacological profiles, simultaneously influencing neurotransmission, ion channel activity, oxidative stress, neuroinflammation, and neuronal survival pathways. Importantly, their biological activity is closely governed by structure–activity relationships (SAR), where physicochemical and stereochemical properties, such as lipophilicity, functional group distribution, and molecular conformation, determine target engagement, BBB permeability, and metabolic stability.

### 3.1. Modulation of GABAergic Neurotransmission

Enhancement of GABA-mediated inhibitory signaling remains one of the most clinically validated strategies for seizure control, and natural products modulate this pathway at multiple levels, including receptor activation, enzyme inhibition, and transporter regulation. Several natural compounds act as positive allosteric modulators of GABA_A_ receptors, enhancing receptor responsiveness without directly activating the receptor. Flavonoids such as baicalein, chrysin, and apigenin interact with the benzodiazepine-binding site, where planar aromatic rings and specific hydroxylation patterns facilitate π–π interactions and hydrogen bonding within the receptor binding pocket. Similarly, neolignans such as magnolol and honokiol exhibit modulatory effects, sometimes via distinct allosteric sites, contributing to subtype-selective activity. SAR studies indicate that substitution patterns on the flavonoid B-ring, along with the presence of a C2–C3 double bond and a 4-keto group, enhance receptor affinity and functional modulation, potentially enabling preferential targeting of GABA_A_ receptor subtypes such as α2/α3 over α1, thereby reducing the sedative side effects commonly associated with classical benzodiazepines [94,95,96]. 

**Figure 2:**
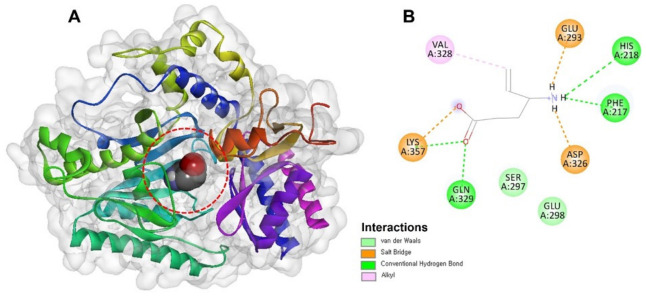
GABA-AT–vigabatrin binding mode and interaction analysis. (**A**) Three-dimensional representation of the protein structure showing the ligand bound within the active site (highlighted by the dashed red circle), illustrating its spatial orientation within the binding pocket. (**B**) Two-dimensional interaction diagram depicting key ligand–residue interactions, including hydrogen bonds, van der Waals contacts, salt bridges, and alkyl interactions with surrounding amino acids, highlighting the residues contributing to binding stability.

In addition to receptor modulation, inhibition of GABA transaminase (GABA-AT), the enzyme responsible for GABA degradation, represents another validated mechanism for increasing synaptic GABA levels. As summarized in Table 1, clinical benchmark inhibition by vigabatrin and representative GABA-AT ligands highlights the importance of GABA-mimetic design and mechanism-based inactivation. In this context, vigabatrin serves as the clinical benchmark for GABA-AT inhibition; it is a structural analogue of GABA and acts as an irreversible (Figure 2), mechanism-based inhibitor of GABA-AT, thereby increasing synaptic GABA levels and producing sustained anticonvulsant effects. However, its use is limited by dose-related adverse effects, most notably visual field toxicity, which highlights the need for a safer, natural-product-inspired alternative [59,97]. Natural-product-inspired scaffolds, including certain flavonoids and alkaloid-like GABA-mimetic structures, have shown moderate GABA-AT inhibitory potential, although their reported activity is generally lower than that of optimized synthetic inhibitors. Electron-rich aromatic systems, conformational restriction, and appropriate stereochemistry can facilitate interaction with the enzyme active site and improve stabilization within the catalytic pocket, supporting further optimization of these chemotypes. 

Furthermore, GABA transporters such as GAT-1, which regulate synaptic GABA reuptake, provide an additional target for modulation [98]. Although most natural products exhibit lower potency compared to synthetic inhibitors, certain scaffolds show potential for optimization, with SAR considerations such as lipophilicity and molecular flexibility playing key roles in influencing transporter binding, membrane permeability, and overall pharmacological efficacy.

**Table 1:** Comparison of natural product inspired GABA-AT inhibitors and vigabatrin.

Compound	Source/Class	Key Binding-Pocket Interactions	Mechanism of Action	Ref.
Vigabatrin	Clinical benchmark; synthetic GABA analogue	Active-site-directed binding as a GABA analogue	Irreversible, mechanism-based inactivation of GABA-AT	[99,100]
3-Amino-1-cyclopentene-1-carboxylic acid	Prototype scaffold relevant to natural-product	Binding depends strongly on stereochemistry and the relative orientation of the amino and carboxylate groups	Competitive inhibition or substrate-like turnover depending on stereochemistry	[101,102]
Cyclohexene GABA analogues	Conformationally restricted vigabatrin analogues	Ring flexibility and correct amino/carboxylate orientation required for active-site-directed inactivation	Mechanism-based inactivation; reversible competitive inhibition	[103]
Pharmacophore screening-based GABA-mimetic natural-product hits	Natural products/Metabolite library hits	Engage residues associated with GABA-like recognition	Putative GABA-AT modulators	[104]
Recent GABA-AT inhibitors	Small-molecule inhibitors	Active-site binding inferred from docking; interactions support recognition of the GABA-AT catalytic pocket	Reversible inhibition for optimized analogues	[105]

### 3.2. Modulation of Glutamatergic Neurotransmission

Reducing excessive glutamatergic excitation complements GABAergic enhancement and is essential for controlling neuronal hyperexcitability in epilepsy. Natural products from diverse chemical classes, including alkaloids, flavonoids, and terpenoids, modulate glutamatergic neurotransmission through multiple mechanisms, particularly via ionotropic and metabotropic glutamate receptors. Recent studies have provided several examples of such activity: hispidulin, a naturally occurring flavone, was reported to inhibit glutamate release from rat cerebrocortical nerve terminals and reduce calcium influx through voltage-gated CaV2.1/CaV2.2 channels; lupeol, a pentacyclic triterpenoid, reduced presynaptic glutamate release and attenuated kainic-acid-induced excitotoxicity in rats; and natural aminosterols were recently shown to inhibit NMDA receptors with low-nanomolar potency, supporting the view that natural scaffolds can directly modulate ionotropic glutamatergic signaling. These compounds exhibit NMDA receptor antagonism or negative allosteric modulation, thereby limiting calcium influx and reducing excitotoxic neuronal damage. SAR studies highlight that features such as aromaticity, the presence of hydrogen-bond donors and acceptors, and precise stereochemical orientation are critical for effective receptor binding and functional modulation. Selective targeting of NMDA receptor subunits, especially GluN2B-containing receptors, is considered advantageous for minimizing adverse effects while preserving therapeutic efficacy. In this context, compounds with moderate lipophilicity and balanced polarity are more likely to achieve optimal central nervous system exposure while reducing off-target interactions [106,107,108]. 

In addition to NMDA receptors, natural products also modulate AMPA and kainate receptors, which are responsible for fast excitatory neurotransmission. Although generally less potent than synthetic antagonists, these compounds provide structurally diverse scaffolds for optimization, where subtle variations in functional groups and stereochemistry can significantly influence receptor affinity and subtype selectivity. Furthermore, metabotropic glutamate receptors (mGluRs), particularly Group II and III subtypes, function as presynaptic autoreceptors that suppress glutamate release when activated. Natural product-derived modulators of these receptors remain relatively underexplored, representing a promising area for the identification of novel scaffolds with improved selectivity and therapeutic potential.

### 3.3. Ion Channel Modulation

Ion channels are central regulators of neuronal excitability, and natural products provide structurally diverse modulators of voltage-gated sodium, calcium, and potassium channels that are relevant to epilepsy management. Natural compounds, particularly alkaloids and terpenoids, can exhibit state-dependent blockade of voltage-gated sodium channels, thereby reducing high-frequency neuronal firing associated with seizure activity. For instance, the aconitum-derived alkaloids lappaconitine and N-desacetyl lappaconitine have been shown to block voltage-gated Na^+^ channels in hippocampal slices and attenuate seizure-like activity induced by low-Mg^2+^ or bicuculline, supporting their role as Na^+^ channel-targeted anticonvulsants. Similarly, marine-derived alkaloids from the sponge genus *Agelas*, such as clathrodin and its analogues, act as state-dependent modulators of Nav1.3, Nav1.4, and Nav1.7 with micromolar-range potency, preferentially binding inactivated channel states, which enhances their therapeutic selectivity. Structural features such as hydrophobic domains and flexible side chains facilitate interaction with channel pore regions and preferential binding to inactivated channel states, enhancing therapeutic selectivity. Targeting specific sodium channel isoforms, such as selective inhibition of Nav1.6 while sparing Nav1.1, represents a promising strategy for improving efficacy while minimizing adverse effects [58,109,110,111]. 

In addition to sodium channels, modulation of calcium channels plays a critical role in controlling neuronal excitability. T-type calcium channels (Cav3.1–3.3) are especially important in absence seizures, whereas N-type and P/Q-type channels regulate presynaptic neurotransmitter release. Natural products, including terpenoids and peptide toxins such as conotoxins, demonstrate activity against these channels; for example, the fungal meroterpenoid ganomycin C has been reported to inhibit low-voltage-gated T-type calcium channels, highlighting the potential of natural scaffolds for absence-seizure-relevant targets. In another recent study, the triterpenoid betulinic acid was shown to preferentially inhibit Cav3.2 and Cav2.2 calcium channels in dorsal-root-ganglion-derived neurons, reducing KCl-evoked Ca^2+^ influx and spontaneous excitatory postsynaptic currents, thereby illustrating a presynaptic, Ca^2+^-channel-dependent mechanism relevant to hyperexcitability and pain, with parallel implications for epilepsy-related circuitry. However, their clinical applicability is influenced by physicochemical properties such as molecular size, polarity, and metabolic stability, with smaller and moderately lipophilic molecules generally offering better translational potential [112,113,114,115,116]. 

Furthermore, activation of potassium channels, including Kv7/KCNQ, BK, and ATP-sensitive (KATP_ATP) channels, represents an effective mechanism for reducing neuronal excitability through membrane hyperpolarization. Natural products capable of modulating these channels often possess structural features that support interaction with channel gating mechanisms, where appropriate lipophilicity and functional group orientation facilitate binding. For example, certain flavonoids and monoterpene glycosides reported in epilepsy-relevant models have been tentatively linked to K^+^-channel-mediated hyperpolarization, although the precise molecular targets are still being characterized. SAR analyses further indicate that electron-donating groups and conformational flexibility contribute to channel activation, highlighting the importance of molecular design in optimizing ion channel-targeting natural products for antiepileptic applications [109,113].

### 3.4. Neuroprotective Mechanisms, Redox Balance, and Pharmacokinetic Considerations

Oxidative stress and neuroinflammation are key contributors to seizure-induced neuronal injury and epileptogenesis, and many natural products exert protective effects through the coordinated modulation of redox balance, inflammatory pathways, and cell survival mechanisms. Polyphenolic compounds, in particular, demonstrate both direct and indirect antioxidant activities, largely attributed to phenolic hydroxyl groups capable of scavenging ROS, with catechol and pyrogallol motifs conferring especially strong radical-scavenging capacity. Recent studies show that flavonoids such as baicalein, hesperidin, and other polyphenol-rich extracts significantly reduce lipid peroxidation, hydrogen peroxide, and nitrite levels while enhancing the activity of endogenous antioxidant enzymes such as superoxide dismutase (SOD), catalase (CAT), and glutathione peroxidase (GPx) in epilepsy-related models. In addition, activation of the Nrf2 signaling pathway enhances endogenous antioxidant defenses by promoting the transcription of cytoprotective genes, a process facilitated by structural features such as electrophilic centers and conjugated systems that enable interaction with the Keap1 regulatory protein. Rosmarinic acid, for example, has been reported to activate the Nrf2/heme-oxygenase-1 (HO-1) axis, reduce superoxide and 4-hydroxynonenal levels, and increase SOD expression in stressed neural and glial models, highlighting its role as a Nrf2-directed endogenous antioxidant booster [117,118,119].

Alongside oxidative stress, neuroinflammation plays a central role in epilepsy progression, and natural products frequently modulate inflammatory signaling pathways. Inhibition of the NF-κB pathway reduces the expression of pro-inflammatory cytokines including IL-1β, IL-6, and TNF-α, while the suppression of cyclooxygenase-2 (COX-2) activity and prostaglandin synthesis further attenuates inflammatory responses. Ferulic acid and rosmarinic acid, for instance, have been shown to downregulate NF-κB, iNOS, and COX-2, decrease IL-1β, IL-6, and TNF-α, and reduce microglial activation and astrogliosis in models of neuroinflammation and excitotoxicity, underscoring their broad anti-inflammatory potential. Additionally, inhibition of the NLRP3 inflammasome limits downstream inflammatory cascades associated with seizure propagation. In several preclinical models, polyphenol-rich extracts and related phytochemicals have been reported to suppress NLRP3 activation and downstream caspase-1-dependent release of IL-1β, which is consistent with their observed anticonvulsant and neuroprotective effects. These anti-inflammatory effects are often governed by structural determinants such as conjugated aromatic systems and reactive functional groups that facilitate interaction with key signaling proteins [118,119,120]. 

**Figure 3:**
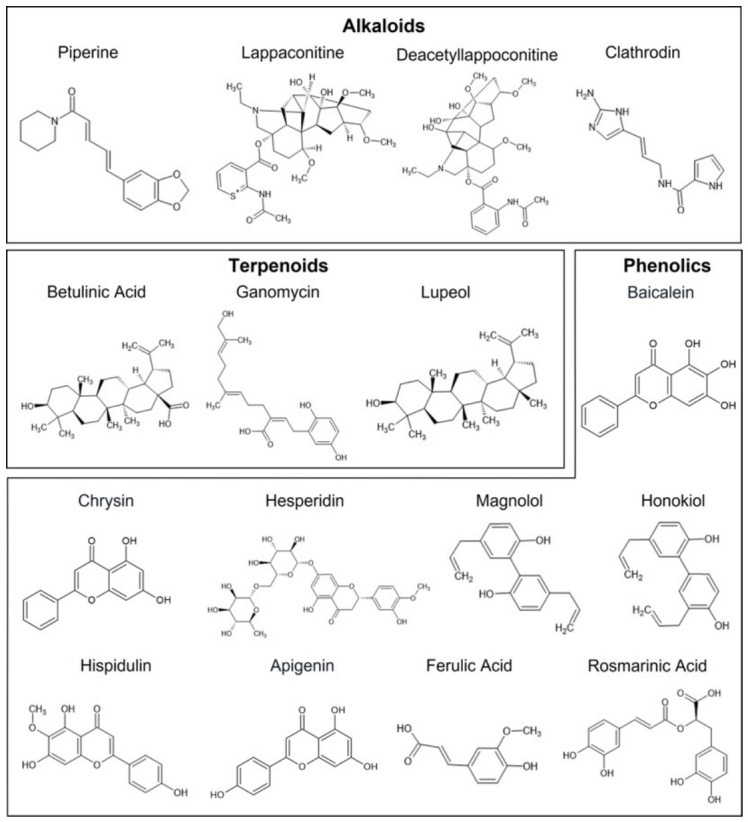
Representative chemical structures of natural compounds with reported antiepileptic or neuroprotective potential, categorized into alkaloids (piperine, lappaconitine, deacetyllappaconitine, clathrodin), terpenoids (betulinic acid, ganomycin, lupeol), and phenolics (baicalein, chrysin, hesperidin, magnolol, honokiol, hispidulin, apigenin, ferulic acid, rosmarinic acid).

Beyond antioxidant and anti-inflammatory actions, natural products also contribute to neuroprotection through mechanisms that preserve neuronal integrity and promote survival. These include the stabilization of mitochondrial function to maintain membrane potential and prevent apoptotic signaling, inhibition of caspase-mediated cell death pathways, activation of pro-survival signaling cascades such as PI3K/Akt and MAPK/ERK, and enhancement of neurotrophic factors, including brain-derived neurotrophic factor (BDNF). Polyphenols such as flavonoids and ferulic acid have been shown to attenuate mitochondrial depolarization, reduce ROS generation, and inhibit caspase-3 and Bax/Bcl-2-related apoptosis in seizure- and ischemia-related paradigms while simultaneously enhancing PI3K/Akt and MAPK/ERK phosphorylation. Rosmarinic acid and other phenolic compounds further upregulate BDNF and TrkB-related signaling in the hippocampus, which is associated with improved synaptic integrity and reduced seizure-associated neuronal loss. Structural features that influence membrane interaction, intracellular targeting, and signaling modulation are critical for these effects, and the emerging evidence from recent preclinical studies suggests that carefully designed polyphenolic scaffolds can simultaneously rebalance redox status, dampen neuroinflammation, and support neuronal survival pathways, offering a multifaceted therapeutic strategy for epilepsy-related neuroprotection (Figure 3) [117,121,122,123]. 

Despite these therapeutic advantages, many natural products face challenges related to pharmacokinetics and safety, including poor bioavailability, limited solubility, rapid metabolism, and, in some cases, toxicity. Alkaloids may exhibit narrow therapeutic windows and dose-dependent neurotoxicity, while flavonoids and other phenolic compounds often suffer from low systemic exposure due to extensive first-pass metabolism (Table 2). Although lipophilicity supports BBB penetration, it may also contribute to bioaccumulation and off-target effects. These limitations highlight the importance of rational optimization strategies, including structural modification, prodrug development, and advanced drug delivery systems, to enhance the efficacy, selectivity, and safety of natural product-based antiepileptic therapies.

## 4. Computational Approaches in Natural Product Discovery for Epilepsy

The integration of computational methodologies has revolutionized natural product drug discovery, enabling the efficient screening of large compound libraries, prediction of pharmacological properties, and rational optimization of lead compounds. For natural product-based epilepsy drug discovery, computational approaches address critical challenges including the vast chemical space of natural products, the requirement for BBB penetration, and the need for multi-property optimization balancing efficacy, safety, and drug-likeness. This section comprehensively examines computational workflows, machine learning applications, and molecular simulation techniques, transforming how natural products are identified, characterized, and optimized as antiepileptic drug candidates.

### 4.1. Natural Product Database Resources

A variety of curated natural product databases support virtual screening and computational drug discovery efforts by providing structurally annotated, and in many cases, biologically characterized compounds. The natural product subset of ZINC Natural Products (within the ZINC database) contains over 200,000 natural and natural product-like compounds in ready-to-dock three-dimensional formats, facilitating structure-based screening workflows. Super Natural II comprises more than 325,000 compounds derived from over 50,000 species and includes calculated physicochemical properties and predicted toxicity profiles [124]. The COCONUT database represents one of the largest open-access repositories, aggregating more than 400,000 unique natural products from multiple public sources. Regionally focused resources further expand accessible chemical diversity. The Traditional Chinese Medicine Database includes over 61,000 compounds from thousands of Chinese medicinal plants, while the African Natural Products Database supports the exploration of African biodiversity [125]. NuBBEDB provides compounds isolated from Brazilian flora and marine organisms. Activity-oriented databases such as NPACT and NPASS link natural products to experimentally reported biological activities and source species, enabling target-focused screening strategies [126]. Additionally, StreptomeDB catalogs metabolites from *Streptomyces* species, a prolific source of bioactive secondary metabolites [127].

These databases provide structural information, calculated molecular properties, source organism data, and where available, biological activity annotations, thereby enabling comprehensive ligand- and structure-based virtual screening campaigns. Nevertheless, database quality and curation standards vary, and preprocessing steps, such as removing duplicates, correcting structural errors, standardizing protonation states, and harmonizing formats, are often required to ensure reliable downstream computational analyses.

### 4.2. Virtual Screening

Virtual screening enables the rapid computational evaluation of large compound libraries to identify molecules with predicted activity against specific therapeutic targets. For natural product-based epilepsy drug discovery, virtual screening approaches leverage both the three-dimensional structures of therapeutic targets and the knowledge of active compounds to prioritize candidates from natural product databases containing hundreds of thousands to millions of compounds.

#### 4.2.1. Structure-Based Virtual Screening and Molecular Docking

Structure-based drug discovery employs three-dimensional structures of biological targets, typically obtained through X-ray crystallography, cryo-electron microscopy, or homology modeling, to predict how small molecules interact with binding sites [128,129]. In this approach, molecular docking algorithms position ligands within the target binding pocket and estimate binding affinity based on predicted protein–ligand interactions. For epilepsy-related targets such as GABA-AT, GABA_A_ receptors, glutamate receptors (NMDA, AMPA, and kainate), and voltage-gated ion channels, available experimental structures or high-quality homology models enable the systematic screening of natural product libraries. Computational docking therefore allows for the rapid identification of compounds with favorable predicted binding, substantially reducing the number of candidates that require experimental testing [130,131].

A comprehensive docking workflow begins with preparing the target structure. High-resolution structures are typically obtained from the protein data bank or generated through homology modeling when experimental data are unavailable. For enzymes such as GABA-AT, multiple crystal structures containing substrates, inhibitors, or cofactors provide valuable information about the active site architecture. Protein preparation generally involves adding hydrogen atoms according to physiological protonation states, assigning correct ionization states to amino acid residues, evaluating crystallographic water molecules to determine whether they participate in ligand interactions, and performing limited energy minimization to remove steric clashes. The binding pocket is then defined based on co-crystallized ligands, mutagenesis evidence, or computational pocket detection algorithms. When homology modeling is required, model quality must be carefully validated, as docking predictions derived from modeled structures typically carry greater uncertainty than those based on experimentally determined structures [132].

Natural product libraries also require extensive preparation before virtual screening. Molecular structures are obtained from curated resources such as ZINC Natural Products, Super Natural II, COCONUT, TCMD, and other specialized databases [133,134,135,136]. Chemical structures are standardized by removing salts, correcting valence states, normalizing tautomers, and assigning appropriate charges. Three-dimensional conformations are generated from two-dimensional representations using conformer generation algorithms, followed by the assignment of physiologically relevant protonation states. Because natural products frequently contain multiple stereocenters, stereochemical variants may be generated when stereochemistry is undefined. Energy minimization is performed to obtain stable initial geometries, and compounds may be filtered according to molecular weight ranges or removal of chemically reactive groups [137]. Molecular descriptors are often calculated for subsequent analysis or machine-learning integration. Although computationally demanding, this preparation step is usually conducted once, allowing prepared libraries to be reused across multiple screening campaigns.

To enable efficient docking calculations, interaction energy grids are generated around the defined binding site. Grid-based methods pre-calculate interaction energies, such as van der Waals, electrostatic, and hydrogen bonding contributions, at discrete spatial points surrounding the pocket. During docking, ligand atoms sample these grids rather than recalculating interactions at each step, which significantly accelerates computation. Accurate binding site definition is essential; grids are typically centered on co-crystallized ligands when available, and the grid dimensions must be large enough to accommodate candidate molecules. Because natural products often possess larger and more flexible structures than conventional drug-like molecules, expanded grid boxes or consideration of multiple potential binding sites may be required [138].

Docking calculations are then performed using specialized algorithms that explore possible ligand orientations and conformations within the binding pocket. Numerous software platforms are available, including Glide, AutoDock Vina, GOLD, DOCK, MOE Dock, and rDock. These programs employ diverse conformational sampling strategies, such as systematic searches, stochastic algorithms, genetic algorithms, or Monte Carlo methods, and utilize different scoring functions derived from empirical, force-field-based, or knowledge-based models. No single docking program consistently outperforms others across all targets, and many studies therefore adopt consensus approaches combining results from multiple algorithms. Screening natural products presents particular challenges due to their structural complexity, increased conformational flexibility, and frequent presence of macrocycles or multiple stereocenters [139,140,141]. Advanced docking strategies such as induced-fit docking, constraint-guided docking, and ensemble docking against multiple protein conformations can improve predictive accuracy by incorporating limited protein flexibility.

Following docking, generated binding poses are scored and ranked according to predicted binding affinity. Scoring functions estimate binding energy by considering several interaction components, including hydrogen bonding, hydrophobic contacts, electrostatic interactions, desolvation effects, and ligand conformational strain. Entropic penalties associated with reduced ligand mobility upon binding may also be approximated. However, docking scores remain imperfect predictors of true binding affinity due to simplified force fields, limited treatment of protein dynamics, and incomplete modeling of solvent and entropic contributions [142]. Consequently, correlations between docking scores and experimental binding data are often moderate. Virtual screening, therefore, focuses primarily on enrichment, identifying a higher proportion of active compounds among the top-ranked candidates rather than accurately predicting absolute affinities.

Post-docking analysis refines the candidate list through additional filtering steps. Visual inspection of top-ranked poses is frequently conducted to ensure chemically plausible binding orientations and appropriate interactions with key residues. Interaction fingerprint analysis can cluster ligands according to shared contact patterns within the binding site, helping identify compounds that may act through similar binding mechanisms. Consensus scoring approaches rescore docking poses with multiple scoring functions to reduce bias from individual scoring models. Drug-likeness and ADMET filtering may also be applied to remove compounds predicted to have poor pharmacokinetic or toxicity profiles, while diversity selection ensures that experimental testing includes chemically diverse scaffolds rather than closely related analogues. Structural similarity comparisons with known active compounds can reveal scaffold-hopping opportunities or validate predicted binding motifs [143,144]. Ultimately, virtual screening yields a prioritized subset of natural products, often dozens to hundreds of candidates, for experimental evaluation.

Application of this workflow to epilepsy targets has proven particularly valuable for enzymes and receptors involved in inhibitory neurotransmission. In the case of GABA-AT, structural studies reveal the presence of the pyridoxal-5′-phosphate (PLP) cofactor within the enzyme’s catalytic site, providing detailed insight into substrate recognition and inhibitor binding. Effective inhibitors typically interact directly with the PLP cofactor, occupy the GABA substrate pocket, and display stereochemical compatibility with the enzyme’s binding geometry. Some inhibitors also extend into adjacent pockets, enhancing binding affinity through additional contacts. Virtual screening of natural product databases against GABA-AT structures can therefore identify molecules capable of forming key interactions within this catalytic environment, which can subsequently be validated through enzymatic inhibition assays [145,146,147,148].

Structure-based virtual screening and molecular docking have been widely used not only for screening, but also for guiding the optimization of natural products and their analogues. For benzodiazepine-site ligands at GABA_A_ receptors, baicalein and related flavones from *Scutellaria baicalensis* were shown to interact with the benzodiazepine binding site in binding studies, supporting the view that hydroxylation patterns can strongly influence receptor affinity. For cannabidiol, recent computational and review studies show that it engages multiple ion-channel and GPCR-related targets, including GPR55 and other receptors, which has motivated structure–activity and target-selectivity studies of CBD-derived analogues [149,150,151,152,153,154,155].

#### 4.2.2. Ligand-Based Virtual Screening

When high-resolution target structures are unavailable, incomplete, or when complementing structure-based approaches, ligand-based methods utilize knowledge of active compounds to identify structurally similar or pharmacophorically related molecules.

Similarity searching is a widely applied ligand-based strategy used to identify compounds that share structural features with known bioactive molecules, based on the similarity–property principle, which suggests that structurally related compounds often exhibit similar biological activities. In this approach, molecular structures are encoded into numerical representations known as fingerprints that describe the presence or absence of specific chemical features or substructures, such as MACCS keys, Daylight fingerprints, or extended-connectivity fingerprints (ECFP). Structural similarity between compounds is typically quantified using statistical coefficients, most commonly the Tanimoto coefficient, although Dice and Cosine metrics are also employed [156]. Compounds within chemical libraries are ranked according to their similarity scores relative to a query molecule, allowing the prioritization of candidates for further screening. Despite its efficiency, similarity searching has limitations, as small structural changes can sometimes produce large differences in biological activity (activity cliffs), and traditional two-dimensional fingerprints may not fully capture three-dimensional conformational or pharmacophoric characteristics [157]. 

Pharmacophore modeling represents another important computational approach for identifying compounds capable of interacting with biological targets. A pharmacophore describes the spatial arrangement of key steric and electronic features necessary for biological activity, independent of a specific molecular scaffold. Common pharmacophoric elements include hydrogen bond donors and acceptors, hydrophobic regions, aromatic rings, and ionizable groups, along with steric constraints that define inaccessible regions within the binding site. Pharmacophore models may be developed using ligand-based approaches, which identify common features among known active compounds, or structure-based approaches derived from protein–ligand complexes that reveal critical interaction points within the binding site. Hybrid models combining both strategies often provide improved predictive capability. During pharmacophore-based virtual screening, large compound libraries are evaluated against the defined spatial and chemical constraints, with multiple conformations generated for each compound to account for molecular flexibility [158,159]. Molecules that satisfy the pharmacophore features are prioritized as potential candidates, facilitating the identification of structurally diverse compounds with similar functional properties.

Similarity-based approaches have likewise been useful for natural-product optimization and scaffold hopping, because fingerprint methods can identify compounds related to known actives even when the scaffolds are not obvious analogues. A recent natural-product similarity-network study used database similarity to highlight natural compounds related to known antibiotics, showing how similarity searching can support compound prioritization in bioprospecting and lead identification. More broadly, a 2021 review noted that virtual screening and cheminformatics methods are now routinely used to explore the chemical diversity of natural products and to prioritize hits for experimental testing, including shape-, fingerprint-, and pharmacophore-based workflows. In one study, ligand-based virtual screening of a large Chinese herbal compound collection identified SIRT1 inhibitors, demonstrating that similarity- and model-based screening can efficiently prioritize natural products for experimental follow-up. Pharmacophore-based virtual screening has also been applied successfully in natural-product-inspired drug design: a review of recent examples highlighted the identification of novel natural inhibitors of *Trypanosoma brucei* glyceraldehyde-3-phosphate dehydrogenase, where pharmacophore screening followed by docking led to five active natural products with sub-30 μM inhibition and two compounds below 8 μM. The same review also described the discovery of lanostane triterpenes as steroid sulfatase inhibitors from natural sources, where ligand-based pharmacophore models retrieved 21 hits from a natural-product database and ultimately yielded three fungal triterpenes with measurable enzyme inhibition [160,161,162].

#### 4.2.3. Quantitative Structure-Activity Relationship (QSAR) Modeling

Quantitative structure–activity relationship (QSAR) modeling establishes mathematical relationships between molecular descriptors and biological activity using statistical or machine learning techniques. Classical QSAR approaches rely on linear regression, multiple linear regression, or partial least squares (PLS), whereas contemporary models increasingly employ machine learning algorithms capable of capturing nonlinear relationships within complex chemical datasets.

In QSAR modeling, chemical structures are first translated into numerical descriptors that encode relevant molecular information. These descriptors may include physicochemical properties such as molecular weight, lipophilicity (logP), polar surface area, and hydrogen bond donor/acceptor counts; topological parameters such as connectivity and shape indices; quantum chemical descriptors including frontier molecular orbital energies and dipole moments; and structural fingerprints or fragment-based features. Using datasets of compounds with experimentally measured activities, the model is trained to learn correlations between these descriptors and biological responses. Successful training requires sufficiently large and chemically diverse datasets, along with reliable and standardized activity measurements [163,164].

Rigorous validation is essential to ensure predictive reliability. Internal validation techniques, such as cross-validation, are complemented by independent test sets and, ideally, external validation datasets to evaluate generalizability. Assessment of the model’s applicability domain further defines the chemical space within which predictions can be considered reliable, based on the coverage and diversity of the training set.

Once validated, QSAR models can be applied in virtual screening campaigns to predict the activity of untested natural products and prioritize candidates with favorable predicted profiles. In one early example, QSAR models built for artemisinin analogues successfully predicted antimalarial activity, and both linear and nonlinear models identified structural features associated with potency, providing a rational basis for modifying the artemisinin scaffold. A later QSAR and docking study on artemisinin derivatives similarly used computational screening to prioritize more active analogues, with several candidates showing very strong predicted antimalarial activity and improved binding interpretations [165,166,167,168]. QSAR has also been applied to flavonoid natural products. A recent study developed a QSAR model for flavonoids as pancreatic lipase inhibitors, then used similarity screening against the COCONUT natural product database to retrieve and prioritize compounds for further docking and ADMET analysis, showing how QSAR can accelerate the discovery of improved natural-product inhibitors. In another natural-product-focused workflow, QSAR-based virtual screening of a compound library identified promising antimalarial hits, and experimental validation confirmed two sesquiterpene lactones with potent activity, demonstrating that QSAR can successfully guide natural-product lead selection before synthesis or testing [169,170].

However, models trained predominantly on synthetic, drug-like molecules may perform suboptimally when applied to structurally complex natural products occupying distinct regions of chemical space [171,172]. Development of natural product-focused QSAR models can help address these limitations and improve predictive performance for natural compound libraries.

### 4.3. Machine Learning and Artificial Intelligence Approaches

Machine learning (ML) and artificial intelligence (AI) methodologies have emerged as transformative tools for predicting molecular properties, optimizing compound structures, and accelerating drug discovery. For natural product-based epilepsy drug discovery, ML approaches address critical prediction tasks including target activity, blood–brain barrier penetration, ADMET properties, and toxicity, enabling data-driven prioritization and optimization.

#### 4.3.1. GNN for Molecular Property Prediction

Graph neural networks (GNNs) have emerged as a transformative approach for molecular property prediction by operating directly on molecular graph representations rather than relying on manually engineered descriptors. In this end-to-end learning paradigm, nodes represent atoms and edges represent chemical bonds, with node features typically encoding atomic number, formal charge, hybridization, aromaticity, number of attached hydrogens, and chirality, while edge features capture bond order, stereochemistry, and conjugation. Graph-level attributes further encode global molecular properties, preserving chemical connectivity and stereochemical information while enabling flexible computational operations on graph structures [41,173,174].

Several GNN architectures have been developed for molecular applications. Graph convolutional networks (GCNs) propagate information between adjacent atoms through graph convolution operations, iteratively aggregating features from neighboring nodes and updating node embeddings using learnable weight matrices and nonlinear activations; deeper layers incorporate information from progressively distant neighbors, enabling the modeling of extended structural patterns and long-range interactions [175,176]. Graph attention networks (GATs) extend this framework by introducing attention mechanisms that assign learned weights to neighboring atoms, allowing the model to emphasize chemically relevant interactions, with multi-head attention further enhancing representational capacity [177]. GraphSAGE (Graph Sample and AggregatE) improves scalability by sampling fixed-size neighbor subsets during aggregation, reducing computational cost while maintaining predictive performance [178].

**Figure 4:**
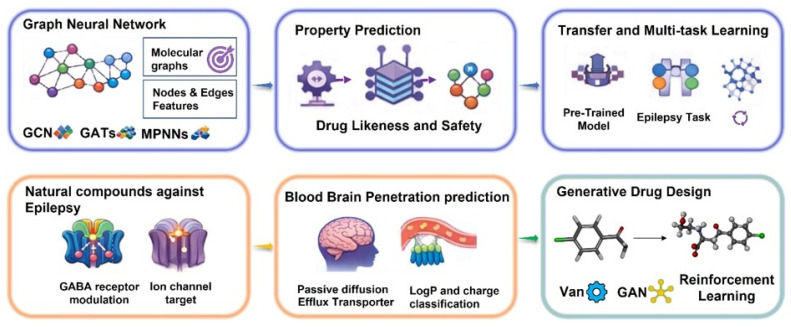
Schematic overview of machine learning and AI-based approaches for epilepsy drug discovery, including molecular graph representation, property prediction for drug-likeness and safety, transfer and multi-task learning, and natural compounds targeting epilepsy-related mechanisms.

Message passing neural networks (MPNNs) provide a unifying framework for many GNN variants, explicitly defining message construction, aggregation, and node update steps (Figure 4). Within an MPNN, messages are generated between connected nodes based on node and edge features, aggregated at each node, and used to update node embeddings iteratively, enabling flexible task-specific architectural design [179]. Directed MPNNs (D-MPNNs), which treat directed bonds rather than atoms as primary entities, have shown advantages in certain molecular property predictions by capturing bond-centric information more effectively. Following message passing, a readout (or pooling) operation aggregates node embeddings into a single graph-level representation via sum/mean pooling, attention-based pooling, or more advanced mechanisms such as Set2Set. The resulting molecular embedding is then passed through multilayer perceptron (MLP) layers to generate property predictions: linear output layers for regression tasks (e.g., binding affinity or logP), and sigmoid/softmax outputs for classification (e.g., BBB penetration) [180].

Training GNN models follows standard supervised learning principles, but poses special challenges in epilepsy-focused and natural-product-oriented work. In epilepsy-driven research, relevant datasets may include GABA-AT inhibition (IC_50_), GABA_A_ receptor modulation, BBB permeability, ADMET parameters, and seizure-related outcomes in preclinical models. However, natural-product databases often lack standardized bioactivity labels (e.g., IC_50_, Ki) compared with large synthetic libraries, resulting in highly imbalanced and sparse data for rare antiepileptic scaffolds. To address this small data problem, transfer learning and self-supervised learning strategies have become increasingly important. Transfer-learning frameworks pretrain GNNs on large, general-purpose compound libraries (e.g., ChEMBL-derived datasets) to learn broad structure–property relationships, then fine-tune on smaller, task-specific natural-product sets, thereby improving target-prediction performance even with limited labeled examples. In parallel, self-supervised molecular-graph pretraining, such as masked-node or masked-substructure prediction, allows models to learn rich structural representations from unlabeled natural-product graphs, which can subsequently be fine-tuned on scarce antiepileptic activity data, thus enhancing generalization and reducing reliance on extensive experimental labeling [181,182,183].

During model training, curated datasets are split to ensure reliable evaluation. While random splitting is common, scaffold-based splitting, where compounds are separated by core chemical scaffolds, provides a more stringent assessment of generalization to novel chemotypes, which is particularly relevant for rare natural scaffolds. Temporal splitting may also be employed when datasets evolve over time, simulating prospective prediction settings. Appropriate loss functions are chosen per task (mean squared error for regression, cross-entropy for classification), and model parameters are optimized using gradient-based algorithms such as Adam, AdamW, or stochastic gradient descent, with validation monitoring to prevent overfitting. Hyperparameter tuning, including depth, hidden dimensions, aggregation functions, learning rate, batch size, and regularization, is typically performed via grid search, random search, or Bayesian optimization. Final performance is evaluated on independent test sets using MAE, RMSE, and R^2^ for regression, and accuracy, precision, recall, F1-score, and ROC-AUC for classification tasks [184].

These methodological components enable GNNs to provide robust, flexible, and scalable frameworks for molecular property prediction, offering significant advantages for computational drug discovery and natural product screening in epilepsy research. GNNs have recently expanded from general molecular property prediction into natural-product-centric drug discovery, where they can learn directly from molecular graphs and support virtual screening, scaffold prioritization, and lead optimization. In one natural-product-focused study, GNN-based models were used to classify natural products from graph representations, and the authors reported better performance than models based on traditional fingerprints, showing that graph learning can capture the structural complexity of natural products more effectively. More importantly for optimization, explainable GNN methods have been shown to provide chemically intuitive fragment-level interpretations that help medicinal chemists identify which substructures drive solubility, genotoxicity, cardiotoxicity, and BBB permeation, thereby supporting structural refinement of candidate molecules [185,186,187,188].

GNNs have also been used in optimization workflows rather than only prediction. A study on graph neural processes for molecules found that graph-based transfer learning outperformed classical single-task and transfer-learning models in docking-score optimization tasks, illustrating how graph models can help prioritize analogues with improved predicted binding profiles. In addition, GNN-based docking frameworks such as MedusaGraph demonstrated that GNNs can generate or rank ligand poses with better speed and accuracy than earlier methods, which is relevant when optimizing natural products that require pose-aware modification. For natural products specifically, a recent review concluded that GNNs may improve outcomes for natural products when used alone or together with standard algorithms, underscoring their growing role in NP-guided lead discovery and modification [189,190].

#### 4.3.2. BBB Penetration and ADMET Properties Prediction 

**Figure 5:**
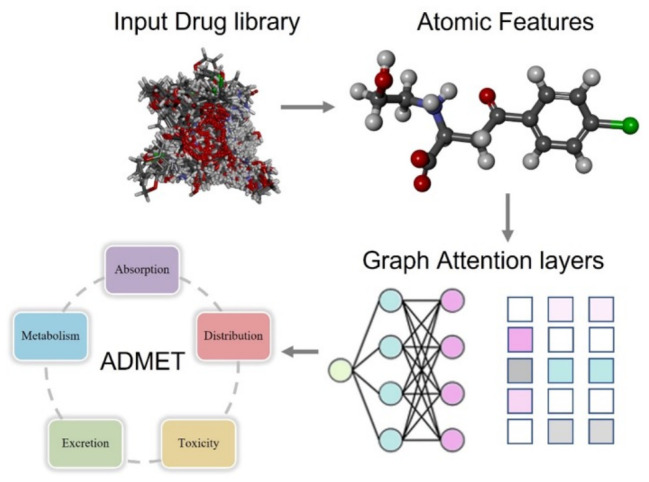
Workflow of a machine learning network for ADMET prediction in drug discovery, showing conversion of the input drug library ADMET (absorption, distribution, metabolism, excretion, and toxicity) properties of each compound.

Accurate prediction of BBB penetration and broader ADMET (absorption, distribution, metabolism, excretion, and toxicity) properties is essential for the successful development of central nervous system therapeutics, as inadequate brain exposure and unfavorable pharmacokinetics remain major causes of clinical failure. BBB permeability, a critical determinant for antiepileptic agents, is governed by a complex interplay of physicochemical and biological factors. Passive diffusion across the BBB is primarily influenced by lipophilicity, molecular size, polar surface area, hydrogen bonding capacity, and molecular flexibility, while active transport mechanisms further regulate brain access. Influx transporters such as LAT1 and OATPs can facilitate compound uptake, whereas efflux transporters, including P-glycoprotein (P-gp) and breast cancer resistance protein (BCRP), significantly restrict central nervous system exposure. In parallel, metabolic stability is required to ensure that compounds remain intact long enough to achieve therapeutically relevant concentrations within the brain. Recent advances in machine learning, particularly GNN-based models trained on experimentally validated datasets, have significantly improved the predictive accuracy for BBB permeability by capturing complex nonlinear structure–property relationships directly from molecular graph representations. These models inherently encode key physicochemical attributes such as molecular weight, logP, polar surface area, aromaticity, and conformational flexibility, which are critical for CNS penetration, and are trained on both binary classification datasets (BBB-penetrant vs. non-penetrant) and quantitative descriptors such as logBB values [191,192]. In the context of natural product screening, early prediction of BBB penetration enables the prioritization of compounds with a higher likelihood of achieving therapeutic brain exposure, thereby improving the efficiency of downstream experimental validation (Figure 5).

Beyond BBB penetration, comprehensive ADMET profiling provides a holistic evaluation of drug-likeness and safety at early stages of drug discovery. Absorption-related properties, including aqueous solubility and intestinal permeability (commonly modeled using Caco-2 or MDCK systems), determine oral bioavailability, while P-glycoprotein substrate prediction informs susceptibility to efflux and reduced systemic exposure. Distribution characteristics, such as plasma protein binding and volume of distribution, influence the free drug fraction and tissue penetration, directly impacting therapeutic efficacy [144,193]. Metabolic considerations focus on cytochrome P450 (CYP)-mediated biotransformation, including the identification of metabolizing isoforms, prediction of CYP inhibition potential, and assessment of drug–drug interaction risks, as well as intrinsic metabolic stability in liver microsomes or hepatocytes. Toxicological predictions further evaluate potential liabilities, including hepatotoxicity, cardiotoxicity, and off-target effects. In the context of natural product-based drug discovery, where compounds often exhibit high structural complexity and unique physicochemical profiles, early integration of BBB and ADMET prediction enables the prioritization of candidates with favorable pharmacokinetic and safety characteristics. This integrated computational framework not only reduces attrition rates in later development stages, but also guides the rational structural modification of natural products to balance CNS exposure, efficacy, and safety [194].

#### 4.3.3. Ensemble GNN Architectures for Enhanced Performance

Individual GNN architectures exhibit complementary strengths and limitations; consequently, ensemble strategies that integrate multiple models often outperform single architectures in molecular property prediction. By combining diverse representational perspectives, ensemble approaches can enhance predictive accuracy, robustness, and generalization.

Several ensemble strategies are commonly employed. In simple averaging (for regression) or majority voting (for classification), multiple GNN models, such as GCN, GAT, GraphSAGE, and MPNN, are trained independently, and their predictions are combined without additional learning. This straightforward method reduces prediction variance and frequently improves overall performance. Weighted averaging extends this concept by assigning higher weights to models demonstrating superior validation performance. Stacking introduces a meta-learner, typically a linear model or shallow neural network, trained to optimally combine predictions from individual GNNs, thereby capturing higher-order relationships among model outputs. Boosting, though less frequently applied to GNNs than to tree-based methods, involves sequential training in which each subsequent model focuses on correcting errors made by its predecessors, potentially improving performance on challenging compounds [195,196,197]. Ensemble models offer multiple advantages. By averaging across diverse architectures, they reduce overfitting and variance, improve generalization by capturing complementary structural patterns, and increase robustness to architectural and hyperparameter choices. Moreover, variability among ensemble predictions can provide a practical estimate of prediction uncertainty; strong agreement across models generally indicates higher confidence.

In the context of natural product-based epilepsy drug discovery, ensemble GNN frameworks can be applied to predict key therapeutic and pharmacokinetic endpoints, including GABA-AT inhibition, GABA_A_ receptor modulation, ion channel interactions, BBB penetration probability, ADMET properties such as solubility and metabolic stability, and toxicity risks including hERG liability or hepatotoxicity. By integrating these predictions into multi-parameter scoring schemes, natural products can be prioritized based on combined activity, central nervous system exposure potential, favorable pharmacokinetics, and low predicted toxicity. Such computational pre-screening substantially enriches the experimental hit rates compared with unguided or random compound selection.

#### 4.3.4. Transfer Learning and Multi-Task Learning

Transfer learning has emerged as an effective strategy for improving predictive performance when task-specific data are limited. In the context of natural product-based epilepsy research, where experimentally validated datasets for specialized targets such as GABA-AT inhibitors or anticonvulsant compounds may comprise only hundreds of examples, transfer learning provides substantial advantages. In this approach, GNN models are first pre-trained on large, diverse molecular datasets (e.g., from ChEMBL or PubChem) to learn generalizable chemical representations. These pre-trained models are subsequently fine-tuned on smaller epilepsy-specific datasets, allowing adaptation of learned molecular features to the target task while retaining broader chemical knowledge [198,199]. This strategy enhances data efficiency and improves performance in low-data regimes typical of niche therapeutic areas. Multi-task learning offers a complementary approach by training a single model to predict multiple related properties simultaneously. In multi-task GNN architectures, initial message-passing layers are shared across tasks, enabling the model to learn general molecular representations. These shared embeddings are then processed through task-specific readout and prediction heads tailored to individual endpoints [200], such as GABA-AT inhibition, BBB penetration, metabolic stability, or toxicity. By sharing lower-level representations while maintaining task-specific output layers, the model benefits from cross-task information sharing without excessive interference between unrelated objectives.

The overall training objective combines losses from each task, typically as a weighted sum, allowing for balanced optimization across endpoints with different scales and importance. Multi-task learning improves data efficiency by leveraging information from all tasks, enhances generalization through shared representations, and mitigates negative transfer by preserving task-specific prediction layers. For natural product screening in epilepsy drug discovery, multi-task GNN models enable the simultaneous prediction of pharmacodynamic activity (e.g., GABA-AT inhibition or GABA_A_ receptor modulation), BBB permeability, key ADMET properties such as solubility and metabolic stability, and major toxicity risks including hERG liability or hepatotoxicity. This integrated, multi-parameter assessment facilitates holistic compound evaluation, reducing reliance on sequential, independent predictions and improving the prioritization of candidates for experimental validation.

#### 4.3.5. Generative Models for De Novo Natural Product-Inspired Design

Generative artificial intelligence models extend computational drug discovery beyond property prediction by enabling the de novo design of novel molecules with predefined characteristics. These approaches allow for the creation of natural product-inspired compounds optimized for epilepsy-related targets and pharmacokinetic requirements. Variational autoencoders (VAEs) learn continuous latent representations of molecular structures by encoding molecules into a compressed latent space and decoding them back into valid chemical structures. Sampling or optimizing within this latent space enables the generation of new molecules with desired predicted properties [201].

Generative adversarial networks (GANs) apply an adversarial framework in which a generator produces candidate molecules while a discriminator evaluates whether the structures resemble real compounds. Through iterative training, the generator learns to produce chemically realistic molecules. Conditional GANs can incorporate property constraints, enabling targeted generation of compounds predicted to exhibit features such as strong GABA-AT inhibition or favorable BBB permeability. Reinforcement learning (RL) approaches treat molecular design as a sequential process in which molecules are constructed stepwise, guided by reward functions that integrate predicted activity, drug-likeness, synthetic feasibility, and safety. Multi-objective optimization enables simultaneous improvement of properties relevant to epilepsy therapy, including potency, CNS exposure, and ADMET characteristics [202].

For natural product-focused discovery, generative models can be trained on natural product databases to capture their structural complexity, stereochemistry, and scaffold diversity. Alternatively, models can retain core natural product scaffolds while optimizing substituents to enhance biological activity or pharmacokinetic properties. To support rational design, interpretability methods such as attention visualization, gradient-based attribution, and substructure perturbation analysis help identify structural features influencing target binding, BBB permeability, or toxicity. Together, these strategies transform AI-based models into practical tools for guiding medicinal chemistry and accelerating the discovery of natural product-inspired antiepileptic agents.

In epilepsy-related discovery, AI-assisted workflows have already been used to analyze large natural-product datasets and prioritize herbal and bioactive compound candidates with evidence of anticonvulsant potential, illustrating how computational models can support rational optimization of natural products for seizure-related indications. Structure-constrained generative methods are also increasingly used to preserve a desirable scaffold while improving drug-like properties; for example, a VAE-RL workflow has been reported to optimize lead compounds for BBB permeability while maintaining key substructures, which is directly relevant to natural product-inspired antiepileptic design [203,204,205].

More generally, generative modeling has been successful in natural-product-inspired optimization by learning chemically realistic latent spaces and then sampling molecules with improved target- or pharmacokinetic-related profiles. Recent AI drug-discovery reviews note that generative models can be trained on large chemical libraries, including natural product space, to generate new molecules with predefined properties and to improve lead molecules through iterative design. For epilepsy-relevant applications, this means that natural product scaffolds such as flavonoids, terpenoids, and alkaloids can be retained as core motifs while substituents are computationally optimized for stronger target engagement, better CNS exposure, and reduced toxicity [204,206].

### 4.4. Molecular Dynamics Simulations

While virtual screening and machine learning identify promising natural product candidates, Molecular Dynamics (MD) simulations provide atomic-resolution insights into dynamic protein–ligand interactions, binding mechanisms, and conformational changes. MD simulations validate static docking predictions, characterize detailed interaction mechanisms, and quantify binding thermodynamics.

#### 4.4.1. MD Simulation Fundamentals

MD simulations model atomic-scale movements over time by numerically integrating Newton's equations of motion:

F = ma = −∇U

where forces (F) derive from potential energy functions (force fields) U encoding bonded interactions (bonds, angles, dihedrals) and non-bonded interactions (van der Waals, electrostatics). Modern force fields for biomolecular simulations (AMBER, CHARMM, GROMOS, OPLS) have been extensively parameterized and validated for proteins, nucleic acids, lipids, and carbohydrates. Small molecule force fields (GAFF, CGenFF) provide parameters for drug-like compounds and natural products [207,208,209].

#### 4.4.2. Simulation Protocol and Application to Epilepsy

MD simulations provide a dynamic and atomistic perspective of protein–ligand interactions, complementing static docking analyses. A standard MD workflow begins with system preparation, typically using a docked complex or experimentally resolved crystal structure as the starting point. Hydrogen atoms are added with appropriate protonation states, and the system is solvated in an explicit water model (e.g., TIP3P or TIP4P). Counterions are introduced to neutralize the system and reproduce physiological ionic strength, and periodic boundary conditions are defined to mimic a bulk environment. Energy minimization follows to remove steric clashes and unfavorable contacts, commonly employing steepest descent and conjugate gradient algorithms. The system is then equilibrated in a stepwise manner. Gradual heating to the target temperature (typically 300 K) is performed under constant volume and temperature (NVT) conditions, followed by constant pressure and temperature (NPT) equilibration to stabilize density [78,209,210,211,212,213,214,215,216,217,218,219,220]. Positional restraints on protein and ligand atoms are initially applied and progressively released to allow controlled relaxation. Production simulations are subsequently conducted without restraints, usually spanning tens to hundreds of nanoseconds, with temperature and pressure maintained using appropriate thermostats and barostats. The resulting trajectories are analyzed to extract structural and energetic information. Trajectory analysis commonly includes root mean square deviation (RMSD) calculations to monitor structural stability, hydrogen bond occupancy to identify persistent interactions, and contact analysis to characterize hydrophobic and other non-bonded interactions. Additional analyses may assess binding pocket geometry, conformational changes, and water-mediated interactions [210,221,222].

In the context of natural product–target interactions, MD simulations are particularly valuable for validating docking predictions. Whereas docking provides a static binding pose, MD reveals whether the ligand remains stably bound or undergoes rearrangement or dissociation. A consistently low ligand RMSD (e.g., <2–3 Å relative to the initial pose) suggests stable binding, whereas large deviations or unbinding events may indicate docking artifacts. For example, natural products predicted to inhibit GABA-AT can be evaluated for sustained interactions within the substrate-binding pocket throughout the simulation.

MD simulations have been used successfully to validate and refine natural product-based inhibitors, guiding their structural optimization in practice. For example, in a structure-based GABA-AT project, plant-derived compounds such as quercetin, salvianolic acid A, and scutellarein were first prioritized by docking, then their binding stability and interaction patterns with GABA-AT were evaluated through multi-nanosecond MD simulations. Low ligand RMSD, persistent hydrogen bonds, and stable hydrophobic contacts in the substrate-binding pocket were used as criteria to select candidates; subsequent cell-based assays confirmed that quercetin and salvianolic acid A inhibited intracellular GABA-AT activity at levels comparable to or better than vigabatrin, validating the MD-guided predictions [223].

In another GABA-AT study, a set of computationally screened inhibitors was subjected to 100 ns MD runs in GROMACS, and RMSD and interaction analyses were used to distinguish compounds that remained stably bound from those that showed significant positional drift or partial dissociation. Here, MD-based ranking helped focus biochemical validation onto a smaller subset of promising candidates, reducing costly experimental screening and directly supporting the selection of compounds with stable pocket occupancy for further development. In parallel, GABA-mimetic compound discovery workflows have used MD to characterize the binding stability of natural-product-like candidates at the GABA_A_ receptor orthosteric site, with very low RMSD and minimal positional fluctuations indicating sustained, high-affinity engagement consistent with strong pharmacological activity [104,147]

#### 4.4.3. Interaction Characterization

MD trajectories also enable the detailed characterization of interaction patterns. Hydrogen bond occupancy analysis distinguishes persistent interactions from transient contacts, helping identify residues critical for binding. Hydrophobic contacts, salt bridges, and π–π stacking interactions can be monitored over time to generate interaction fingerprints that highlight binding hot spots. Such comprehensive mapping supports the rational interpretation of SARs and guides optimization strategies [210,224,225].

Importantly, MD simulations capture induced fit effects and conformational flexibility that static models cannot represent. Binding site residues may rearrange to accommodate ligands, loops may modulate pocket accessibility, and allosteric conformational changes may occur in distal regions. These dynamic insights are particularly relevant for flexible targets such as GABA_A_ receptors or ion channels, where ligand binding may influence channel gating or receptor modulation.

Explicit solvent modeling further allows for the investigation of water-mediated interactions. Conserved water molecules may bridge proteins and ligands through hydrogen-bond networks, contributing significantly to binding stability. MD simulations can identify such persistent waters, assess their stability, and inform whether ligand modifications should aim to displace or retain them [226]. Collectively, MD simulations provide a robust framework for validating and refining natural product–target interactions, offering mechanistic insights that enhance computational drug discovery efforts in epilepsy research.

### 4.5. Free Energy Calculations

Quantitative binding affinity predictions enable the prioritization of natural products and optimization of derivatives.

#### 4.5.1. Free Energy Perturbation (FEP)

Free energy perturbation (FEP) calculations provide a rigorous framework for estimating relative binding free energies between structurally related ligands through alchemical transformations. In this approach, ligand A is computationally transformed into ligand B via a series of nonphysical intermediate states [227]. The relative binding free energy difference is defined as:

ΔΔG_bind_ = ΔG_bind(B)_ − ΔG_bind(A)_

Using a thermodynamic cycle, simulations are performed for both ligands in the protein-bound state and in solution, yielding:

ΔΔG_bind_ = ΔG_protein A→ B_ − ΔG_solution A→ B_

This formulation enables quantitative prediction of how structural modifications influence binding affinity. FEP is particularly valuable in medicinal chemistry optimization, as it can assess the energetic consequences of functional group substitutions, methylation, stereochemical inversion, or scaffold modifications [227,228]. For example, it can predict whether methylating a hydroxyl group enhances GABA-AT binding, how stereochemistry affects affinity, or which substituents optimize potency. Although computationally demanding, requiring multiple well-converged simulations per transformation, properly executed FEP calculations can achieve high accuracy, often within approximately 1 kcal/mol of the experimental measurements, making them a powerful tool for rational lead optimization [229].

#### 4.5.2. MM/GBSA and MM/PBSA

Molecular mechanics/generalized Born surface area (MM/GBSA) and molecular mechanics/Poisson–Boltzmann surface area (MM/PBSA) methods provide computationally efficient approaches for estimating binding free energies through the post-processing of molecular dynamics trajectories. The binding free energy is commonly expressed as:

ΔGbind = ΔE_MM_ + ΔG_solv_ − TΔS

where ΔE_MM_ represents the molecular mechanics energy (van der Waals and electrostatic contributions), ΔG_solv_ corresponds to the solvation free energy calculated using implicit solvent models (generalized Born or Poisson–Boltzmann) with an additional surface area term, and TΔST\Delta STΔS denotes the entropic contribution, often approximated using normal mode analysis [230,231,232]. Although MM/GBSA and MM/PBSA are generally less accurate than free energy perturbation methods, with typical errors in the range of 2–3 kcal/mol, they are substantially less computationally demanding. Consequently, they are widely used for the rapid ranking and prioritization of compounds rather than precise quantitative affinity prediction [59,211,223].

#### 4.5.3. Enhanced Sampling and Metadynamics

Conventional molecular dynamics simulations are typically limited to nanosecond–microsecond timescales, which may be insufficient to capture slow conformational transitions, ligand binding or unbinding events, and other rare processes. Enhanced sampling techniques address these limitations by accelerating exploration of the conformational landscape [233].

Metadynamics is a widely used enhanced sampling approach in which history-dependent bias potentials are applied along selected collective variables (CVs). By progressively discouraging the system from revisiting previously sampled states, these biasing potentials effectively “fill” free energy minima, enabling the system to escape local energy basins and explore new conformations [234,235]. 

Studies on flexible protein targets have also used metadynamics with path collective variables to map conformational transitions and ligand-binding routes, which is especially valuable when natural products bind through multiple orientations or trigger induced-fit effects. More generally, metadynamics simulations of ligands binding to protein surfaces demonstrated that the method can recover experimentally consistent binding poses even when conventional pocket-based docking is challenging, supporting its use in natural-product optimization campaigns [236,237,238]. This strategy is particularly valuable for investigating ligand binding pathways, sampling multiple binding modes, and overcoming high-energy barriers associated with conformational transitions, thereby providing a more comprehensive characterization of protein–ligand interactions than standard MD simulations alone.

#### 4.5.4. Replica Exchange and Accelerated MD

**Figure 6:**
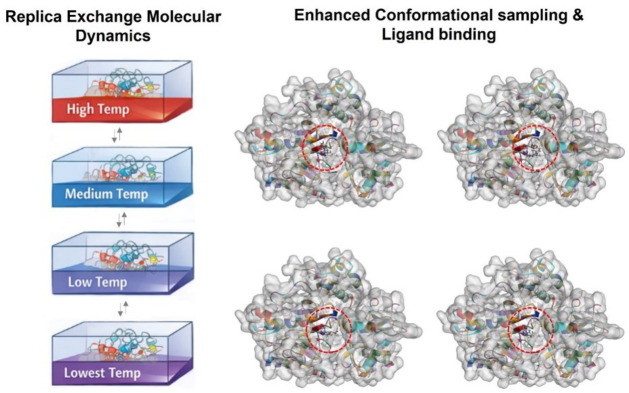
Schematic illustration of replica exchange molecular dynamics (REMD) used to enhance conformational sampling and ligand binding by exchanging simulations across high, medium, low, and lowest temperature replicas.

Replica exchange molecular dynamics (REMD) enhances conformational sampling by running multiple simulations (replicas) in parallel at different temperatures, with periodic attempts to exchange configurations between replicas (Figure 6). High-temperature replicas facilitate the crossing of energy barriers, whereas low-temperature replicas sample thermodynamically relevant conformations [239]. This exchange mechanism improves exploration of the conformational landscape and reduces trapping in local minima. REMD is particularly useful for sampling protein conformational ensembles, exploring alternative ligand binding modes, and refining docking-derived complexes.

Accelerated Molecular Dynamics (aMD) employs a modified potential energy surface in which energy wells are selectively raised while high-energy regions remain largely unaffected. This smoothing of energy barriers promotes more frequent transitions between conformational states, effectively extending accessible simulation timescales by one to two orders of magnitude [240]. aMD is therefore well-suited for sampling large-scale conformational changes, investigating dynamic binding processes, and capturing rare events such as ligand unbinding. REMD and aMD have been successfully applied to sample multiple protein conformations and ligand-bound states, elucidate conformational transitions coupled to ligand binding, and accelerate rare-event sampling in serine proteases, ribose-binding proteins, and amyloidogenic peptides, illustrating their utility for refining docking-derived complexes and exploring alternative binding modes of natural product-like ligands [241,242,243,244].

## 5. Translational Challenges and Future Perspectives

Despite the tremendous potential of natural products for epilepsy treatment, significant challenges impede translation from bench to bedside. Addressing these challenges requires multidisciplinary approaches integrating traditional knowledge, modern pharmacology, computational prediction, medicinal chemistry, formulation science, and clinical investigation.

### 5.1. Pharmacokinetic Barriers

#### 5.1.1. Blood–Brain Barrier (BBB) Penetration

The BBB, formed by tightly connected endothelial cells of brain capillaries, acts as a selective interface that protects neural tissue while restricting the entry of many therapeutic compounds. Effective BBB penetration is influenced by molecular properties such as molecular weight (<400–500 Da), moderate lipophilicity (logP ~1.5–3.5), limited hydrogen bonding, and low polar surface area (<90 Å^2^). In addition to passive diffusion, transporter systems also regulate brain entry. Efflux transporters, including P-glycoprotein (P-gp), BCRP, and MRPs, reduce drug accumulation in the brain, whereas certain influx transporters may facilitate uptake of specific compounds [245].

To improve BBB penetration, several strategies have been explored. Structural optimization can adjust physicochemical properties to favor diffusion, while prodrug approaches temporarily mask polar groups to enhance brain entry. Nanoparticle-based delivery systems such as liposomes and polymeric nanoparticles can also improve brain targeting. More advanced techniques, including focused ultrasound-mediated BBB disruption, may further enhance drug delivery to the central nervous system.

#### 5.1.2. Computational Prediction and Optimization

Machine learning models predicting BBB penetration from the molecular structure enable the early identification of natural products likely to achieve adequate brain exposure. GNN models and other ML approaches trained on experimental BBB data can screen virtual libraries, prioritizing compounds with favorable predicted penetration. For compounds with suboptimal predicted BBB penetration but promising activity, computational models guide structural modifications to improve brain entry while maintaining target activity [246]. This in silico optimization reduces experimental iterations required for developing CNS-penetrant derivatives.

Recent advances have extended this paradigm from simple structure–property prediction to constrained generative optimization, where deep generative models simultaneously optimize antiepileptic potency and BBB permeability under predefined structural constraints. Variational autoencoders (VAEs) and graph-based generative models, for example, can encode molecular structures into a continuous latent space and then sample or perturb molecules under multi-objective reward functions that jointly maximize predicted target engagement (e.g., GABA-AT inhibition or GABA_A_ receptor modulation) and BBB permeability while penalizing undesirable properties such as toxicity or excessive lipophilicity. In one structure-constrained molecular generation workflow for central nervous system targets, a VAE-based framework integrated with reinforcement-learning-style optimization successfully enhanced BBB permeability while preserving key pharmacophoric motifs, demonstrating the feasibility of balancing cross-barrier exposure with target-specific activity [205,247]. 

These generative approaches can be further coupled with fast BBB-penetration predictors (e.g., deep-learning or graph-based models trained on large in vitro or in silico datasets) to rapidly evaluate candidate structures and steer the optimization process toward molecules that satisfy competing objectives. By encoding BBB-related constraints, such as polar surface area, logP, hydrogen-bond patterns, and efflux-transporter liability, into the reward or loss function, such methods effectively perform BBB-aware scaffold engineering, prioritizing derivatives that are both brain-penetrant and antiepileptically active. This paradigm not only accelerates the design of CNS-targeted natural-product derivatives, but also shifts BBB optimization from a post hoc correction step to an integral, mechanism-informed component of antiepileptic drug discovery.

#### 5.1.3. Bioavailability and First-Pass Metabolism

Many natural products, particularly polyphenolic compounds, exhibit poor oral bioavailability due to limited aqueous solubility, low intestinal permeability, and extensive first-pass metabolism. Phase II conjugating enzymes, including UDP-glucuronosyltransferases, sulfotransferases, and catechol-O-methyltransferases, expressed in intestinal enterocytes and hepatocytes, rapidly convert these compounds into glucuronidated, sulfated, or methylated metabolites. This first-pass effect substantially reduces systemic exposure to the parent molecule, although certain metabolites may retain partial biological activity [248].

Multiple strategies have been explored to improve bioavailability. Formulation-based approaches, such as solid dispersions, self-emulsifying drug delivery systems, cyclodextrin complexation, and nanoformulations, enhance solubility and absorption. Co-administration with permeability enhancers (e.g., piperine) can transiently increase intestinal uptake. Prodrug strategies, including lipophilic ester or ether derivatives, aim to bypass rapid conjugation and release the active compound after absorption. Structural optimization to block metabolic hotspots, such as methylation of phenolic hydroxyl groups to reduce glucuronidation, can further improve metabolic stability. In some cases, alternative administration routes, including parenteral, transdermal, or intranasal delivery, are employed to circumvent first-pass hepatic metabolism, although these approaches introduce additional formulation and compliance considerations [249].

### 5.2. Standardization and Quality Control

Unlike synthetic drugs with defined structures and controlled manufacturing, natural product preparations such as crude extracts or mixtures often show significant batch-to-batch variability due to differences in plant genetics, cultivation conditions, harvesting, storage, and extraction methods. This variability presents challenges for clinical development and regulatory approval. Standardization requires detailed chemical characterization using techniques such as HPLC, mass spectrometry, and NMR, along with quantification of marker compounds to ensure consistency. Botanical authentication and contaminant screening for heavy metals, pesticides, and microbes are also essential for quality control. Implementation of Good Agricultural and Collection Practices (GACP) and Good Manufacturing Practices (GMP) further improves reproducibility [250]. While regulatory pathways exist for complex botanical preparations, single-compound natural products generally provide clearer regulatory frameworks and more predictable pharmacological profiles.

### 5.3. Safety and Toxicity Considerations

While natural products are often perceived as inherently safe due to long traditional use, this perception can be misleading. Natural products can exhibit significant toxicity, and traditional use often lacks the rigorous safety evaluation required for modern pharmaceuticals. Comprehensive preclinical toxicology studies (acute toxicity, repeated-dose toxicity, genotoxicity, reproductive toxicity, carcinogenicity, as appropriate) are essential. Special attention to organ-specific toxicities (hepatotoxicity, nephrotoxicity, cardiotoxicity) is warranted as many natural products undergo hepatic metabolism and renal excretion [251].

Herb–drug interactions represent important safety concerns. Natural products may inhibit or induce cytochrome P450 enzymes, affecting the metabolism of co-administered drugs [252]. Transporter interactions can alter the absorption, distribution, and elimination of other medications. For epilepsy patients often receiving polytherapy, interaction potential requires careful evaluation. Long-term safety, particularly important for chronic epilepsy treatment, requires extended preclinical and clinical studies. Some natural products exhibit cumulative toxicity or delayed adverse effects not apparent in short-term studies.

### 5.4. Clinical Development Challenges

Natural product drug development is subject to the same rigorous clinical trial requirements as synthetic pharmaceuticals, including dose-escalation studies, randomized placebo-controlled efficacy trials, and comprehensive long-term safety evaluations. Despite this shared regulatory standard, several challenges are particularly pronounced for natural products. Intellectual property protection remains a major limitation, as naturally occurring compounds in their unmodified form are generally not patentable. Although specific derivatives, novel formulations, manufacturing processes, or new therapeutic indications may be protected, the comparatively narrow scope of exclusivity can reduce commercial incentives for costly clinical development. Regulatory pathways for botanical drugs and complex mixtures are also less straightforward than those for single, well-defined synthetic entities [253]. While regulatory agencies have issued guidance frameworks for botanical products, each submission often requires case-specific evaluation and extensive characterization [253].

Funding constraints further complicate development, as natural product research frequently depends on academic institutions, government grants, or small biotechnology companies rather than large pharmaceutical investment. Additionally, cultural, legal, and political considerations may influence regulatory evaluation and market access, particularly for products subject to legal restrictions, sustainability concerns, or complex supply chains.

## 6. Conclusions

Natural products represent an evolutionarily optimized source of chemical diversity with significant potential for epilepsy therapy, particularly for the 30–40% of patients with drug-resistant epilepsy. Major classes such as alkaloids, terpenoids, and phenolic compounds exhibit diverse pharmacological activities, including the modulation of GABAergic and glutamatergic signaling, ion channel regulation, antioxidant and anti-inflammatory effects, and neuroprotection. Their multi-target properties may be advantageous in addressing the complex and multifactorial mechanisms underlying epileptic disorders.

Advances in computational approaches have substantially accelerated natural product-based drug discovery. Techniques such as virtual screening, molecular docking, and machine learning, especially GNN, enable the rapid prediction of target affinity, BBB permeability, and ADMET properties. In addition, Molecular Dynamics simulations and binding free energy calculations provide mechanistic insights that support rational optimization of promising compounds. Although challenges remain, including pharmacokinetic limitations, BBB penetration, and compound standardization, successful examples such as cannabidiol (Epidiolex) demonstrate the feasibility of translating natural products into antiepileptic therapies. Integrating ethnopharmacological knowledge with advanced computational and experimental strategies offers a promising pathway for the discovery and development of novel antiepileptic agents.

## Figures and Tables

**Table 2:** Predicted physicochemical properties and BBB permeability comparison of the discussed natural compounds calculated using SwissADME.

Name	MW (g/mol)	LogP	TPSA (A^2^)	HBD	HBA	BBB Permeability
**Alkaloids**						
Piperine	285.34	3.03	38.77	0	3	Yes
Lappaconitine	584.70	1.87	126.79	3	9	No
Deacetyllappoconitine	600.70	1.36	147.02	4	10	No
Clathrodin	231.25	0.61	99.59	4	2	No
**Terpenoids**						
Betulinic acid	456.70	6.14	57.53	2	3	No
Ganomycin	360.44	3.60	97.99	4	5	No
Lupeol	426.72	7.27	20.23	1	1	No
**Phenolic compounds**						
Baicalein	270.24	2.24	90.90	3	5	No
Chrysin	254.24	2.55	70.67	2	4	Yes
Apigenin	270.24	2.11	90.90	3	5	No
Magnolol	266.33	4.25	40.46	2	2	Yes
Honokiol	266.33	4.19	40.46	2	2	Yes
Hispidulin	300.26	2.12	100.13	3	6	No
Hesperidin	610.56	−1.06	234.29	8	15	No
Ferulic acid	194.18	1.36	66.76	2	4	Yes
Rosmarinic acid	360.31	1.58	144.52	5	8	No

## Data Availability

No new data were created or analyzed in this manuscript. Data sharing is not applicable to this article.
